# New steroidal aromatase inhibitors: Suppression of estrogen-dependent breast cancer cell proliferation and induction of cell death

**DOI:** 10.1186/1471-2121-9-41

**Published:** 2008-07-24

**Authors:** Margarida Cepa, Georgina Correia-da-Silva, Elisiário J Tavares da Silva, Fernanda MF Roleira, Margarida Borges, Natércia A Teixeira

**Affiliations:** 1 Biochemistry Laboratory, Faculty of Pharmacy, University of Oporto, Rua Aníbal Cunha, 164, 4099-030 Oporto, Portugal; 2 IBMC – Institute for Molecular and Cellular Biology, University of Oporto, 4150-180 Oporto, Portugal; 3 Centro de Estudos Farmacêuticos, Pharmaceutical Chemistry Laboratory, Faculty of Pharmacy, University of Coimbra, 3000-295 Coimbra, Portugal

## Abstract

**Background:**

Aromatase, the cytochrome P-450 enzyme (CYP19) responsible for estrogen biosynthesis, is an important target for the treatment of estrogen-dependent breast cancer. In fact, the use of synthetic aromatase inhibitors (AI), which induce suppression of estrogen synthesis, has shown to be an effective alternative to the classical tamoxifen for the treatment of postmenopausal patients with ER-positive breast cancer. New AIs obtained, in our laboratory, by modification of the A and D-rings of the natural substrate of aromatase, compounds **3a **and **4a**, showed previously to efficiently suppress aromatase activity in placental microsomes. In the present study we have investigated the effects of these compounds on cell proliferation, cell cycle progression and induction of cell death using the estrogen-dependent human breast cancer cell line stably transfected with the aromatase gene, MCF-7 aro cells.

**Results:**

The new steroids inhibit hormone-dependent proliferation of MCF-7aro cells in a time and dose-dependent manner, causing cell cycle arrest in G_0_/G_1 _phase and inducing cell death with features of apoptosis and autophagic cell death.

**Conclusion:**

Our *in vitro *studies showed that the two steroidal AIs, **3a **and **4a**, are potent inhibitors of breast cancer cell proliferation. Moreover, it was also shown that the antiproliferative effects of these two steroids on MCF-7aro cells are mediated by disrupting cell cycle progression, through cell cycle arrest in G_0_/G_1 _phase and induction of cell death, being the dominant mechanism autophagic cell death. Our results are important for the elucidation of the cellular effects of steroidal AIs on breast cancer.

## Background

A large proportion of breast cancer patients are postmenopausal women with estrogen receptor-positive (ER) tumors. After menopause, the main source of circulating estrogens are extragonadal sites, such as liver, skin, muscle and adipose tissue [1-3]. Recent advances in treatment strategies, that inhibit the action of estrogen, have greatly improved the range of effective therapeutic options for breast cancer in postmenopausal women. In fact, hormonal therapies have shown to be important tools in treating ER-positive breast cancer and during the last two decades, tamoxifen, which blocks the action of estrogen via the ER, has been considered the gold standard therapeutic option [4]. However, extensive evaluation of tamoxifen treatment revealed adverse effects such as endometrial cancer and blood clots. In addition, many ER-positive breast cancers do not respond to this therapeutic and resistance to tamoxifen often develops during treatment, leading to disease recurrence [5-7]. To circumvent these drawbacks the use of third-generation aromatase inhibitors (AIs), which prevent estrogen biosynthesis, is an effective alternative hormonal therapy and clinical guidelines are now embracing AIs as appropriate adjuvant therapy for hormone-sensitive early breast cancer [8,9]. These compounds have demonstrated superior efficacy, reduced incidence of endometrial cancer and blood clot formation when compared to tamoxifen. Moreover, AIs have also improved disease-free survival in a variety of adjuvant settings for early breast cancer [9,10]. Steroidal and non-steroidal AIs cause an effective suppression of estrogen synthesis [11,12]. The former, such as exemestane and formestane, compete with the endogenous ligands, androstenedione and testosterone, for the active site of the aromatase and are converted to intermediates that bind irreversibly to the enzyme active site. Non-steroidal AIs, like letrozole and anastrazole, bind reversibly to the enzyme active site, competing with the substrate of aromatase. Despite the success of the third-generation steroidal and nonsteroidal AIs, they also induce increased bone loss, which may heighten the risk for osteoporotic fractures and bone pain. In that way, it is essential to search for other potent and specific molecules with lower side effects. Moreover, it is of critical importance for the management of breast cancer treatment to understand the pathways involved in the regression of breast tumors by AIs.

For many years, research in the field of endocrine-mediated breast cancer has focused on the proliferative effects of estrogens. However, recent work has also demonstrated a role for these steroidal hormones in the regulation of apoptosis in neoplastic mammary tissue and in breast cancer cell lines [13,14]. On the other hand, it has been reported that estrogen stimulates the growth of breast cancer expressing functional ERs [15-17], by affecting cell cycle machinery [18,19] and inducing expression of specific growth factors and their receptors [20,21]. It has been reported that estradiol deprivation [22] or treatments with selective estrogen receptor modulators (SERMs) [23-26], antagonists of estrogen receptor [27] or aromatase inhibitors [28] induce inhibition of cell proliferation and apoptosis in breast cancer cells. Treatment of breast cancer using these endocrine strategies may induce cell death by altered expression of Bcl-2 family proteins, altered expression of cell cycle associated proteins [13,27,28] or by other mechanisms.

**Figure 1:**
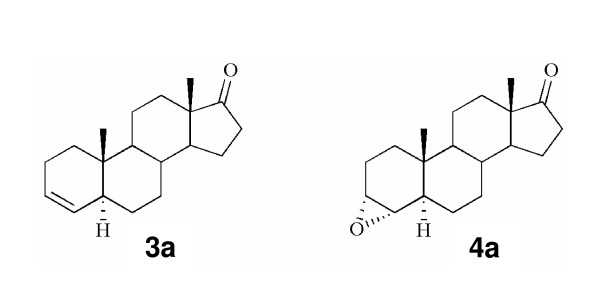
Structures of the 3-deoxy steroid 3a and its 3,4-epoxide derivative 4a.

New synthetic AIs obtained by modifications in the A and D- rings of the natural substrate of aromatase, androstenedione were developed and tested in our laboratory, for aromatase inhibition in placental microsomes. Two of the compounds synthesized, the 5α-androst-3-en-17-one (**3a**) and the 3α ,4α-epoxy-5α-androstan-17-one (**4a**) (Figure 1), have shown to be strong inhibitors of aromatase [29]. In the present study, we have investigated the effects of these two steroidal aromatase inhibitors on cell proliferation, cell cycle progression and induction of cell death, using the estrogen-dependent human breast cancer cell line stably transfected with the aromatase gene, MCF-7aro.

## Results

### Morphological studies

**Figure 2:**
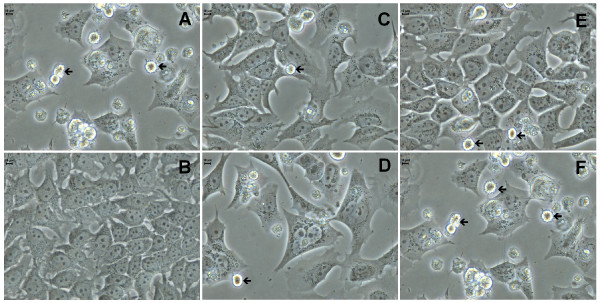
**Morphological changes of MCF-7aro cells treated with compounds 3a and 4a**. (A) hormone depleted medium, (B) untreated cells, or treated for 72 hr with 3a (C-D) and 4a (E-F), respectively with 10 and 25 μM. Arrows indicate multiblebbing cells and arrowheads indicate perinuclear vesicles in the cytoplasm.

**Figure 3:**
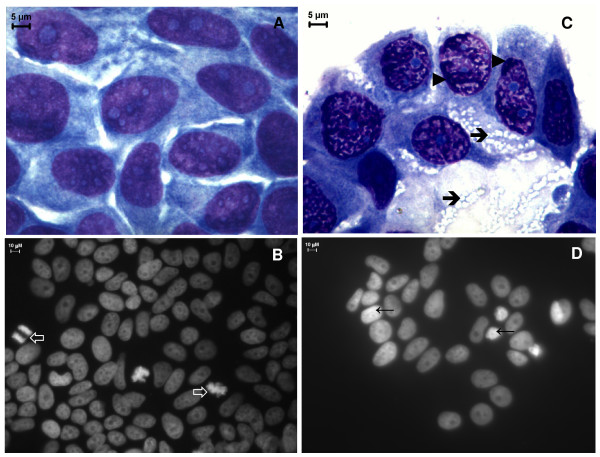
**Effects of compounds 4a on cell morphology**. Cultured MCF-7aro cells were examined in the absence (A, B) or in the presence of 25 μM of 4a (C, D) in a medium containing 1 nM T for 72 hr. Wright staining shows that cells treated with 4a have condensed and marginalized chromatin (arrowheads) and cytoplasm vacuolization (arrows) (C) in comparison to the control cells (A). Nuclear morphological changes in MCF-7aro cells were demonstrated by Hoechst 33258 staining under the fluorescence microscope. Untreated cells exhibited normal nuclear morphology and the presence of abundant mitotic figures (open arrows) (B). Treatment with 4a induced chromatin condensation (arrows) (D).

In order to investigate the morphological changes induced by inhibitors **3a **and **4a **in MCF-7aro cells, cells were cultured with or without the compounds and examined by phase contrast microscopy (Figure 2). The untreated control cells (medium with 1 nM T) did not show any morphological alterations during the different incubation periods. After 24 hr incubation with the different concentrations of the two inhibitors no significant differences in cell and nuclear morphology were observed. However, after 3 and 6 days, MCF-7aro cells treated with compounds **3a **and **4a **showed marked morphological alterations. In treated MCF-7aro cells we could observe non-adherent round cells, showing membrane blebbing, as well as adherent cells characterized by the presence of perinuclear vesicles in the cytoplasm (Figure 2). When exposed to the higher doses of the compounds, an increased number of these vesicles were observed in the cells. Additionally, Wright and Hoechst 33258 staining (Figure 3) confirmed the cellular changes upon treatment with the compounds. Treatment of MCF-7aro cells with higher concentrations of **3a **and **4a **(25 and 50 μM) resulted in dramatic alterations in cellular morphology with condensed marginalized chromatin and vacuolization of the cytoplasm.

### Cell viability and cell proliferation

**Figure 4:**
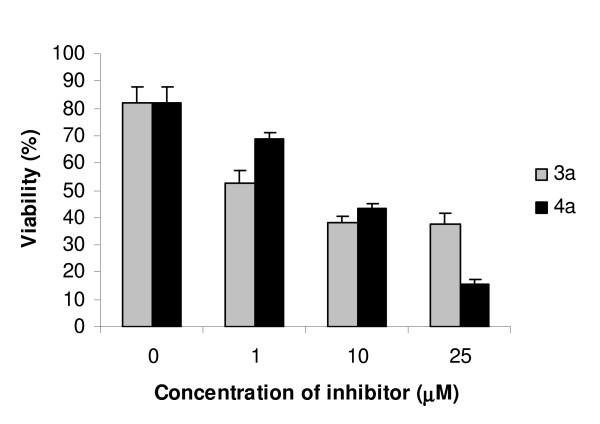
**Effects of 3a and 4a on cell viability**. MCF-7aro cells treated with 3a and 4a induced a decrease in 7AAD-ve cells, in comparison to control. Data are expressed as the percentage of 7AAD-ve cells and are representative of triplicate cultures and three independent experiments.

**Figure 5:**
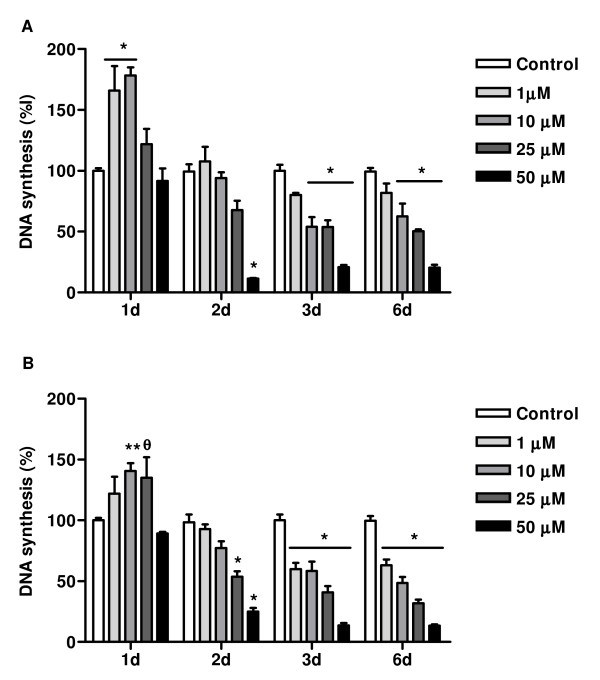
**Effects of inhibitor 3a (A) and 4a (B) on rate of DNA synthesis**. MCF-7aro cells were cultured in steroid-free medium without phenol red for 3 days before plating. Triplicate wells were treated with the indicated concentrations of the compounds in medium containing 1 nM T. Cells cultured with testosterone represented maximum cell proliferation and were considered as control. 3a and 4a induced a decrease in cell proliferation, evaluated by the thymidine incorporation assay, in a time- and dose-dependent manner. Results are the mean ± SE of three independent experiments whereas cultures were performed in triplicate. Significant inhibition relative to the control level is denoted by * (*P *< 0.001), ** (P < 0.01) and θ (P < 0.05).

A significant dose- and time-dependent reduction in cell number was observed when cells were incubated with compounds **3a **and **4a**. Cell viability was evaluated by flow cytometry using 7AAD- fluorescence. 7AAD-ve cells were considered as viable cells. Treatment of MCF-7aro cells with compound **3a **for 72 hr, resulted in a reduced number of 7AAD-ve cells: 29.4 ± 7.1%, 43.6 ± 1.0% and 44.3 ± 0.4%, respectively for 1, 10 and 25 μM, whereas compound **4a **reduced cell viability in 13.1 ± 3.1%, 38.4 ± 3.7% and 66.6 ± 1.2%, respectively for 1, 10 and 25 μM, in comparison to the control (Figure 4). To address the direct effect of aromatase inhibitors **3a **and **4a **on the proliferation of MCF-7aro cells, thymidine incorporation assays were performed. For this purpose, exponentially growing cells were treated with different concentrations (1–50 μM) of the inhibitors, **3a **and **4a**, for 1–6 days. As shown in Figure 5, these compounds inhibited MCF-7aro cell growth in a dose- and time-dependent manner. The time-response profile obtained for both inhibitors showed a biphasic effect in DNA synthesis of MCF-7aro cells. At short exposure times, compounds **3a **and **4a **stimulated DNA synthesis of MCF-7aro cells. In fact, after 24 hr incubation, compound **3a **induced 66 ± 20%, 78 ± 7% and 22 ± 13% and compound **4a **induced 22 ± 14%, 41 ± 6% and 35 ± 17% increase in DNA synthesis, respectively for 1, 10 and 25 μM, considering the control as 100%. For 24 hr incubation of MCF-7aro cells with **3a **and **4a **at 50 μM, a slight decrease in thymidine incorporation was observed. After this period, when cells were treated for 3 and 6 days with these compounds, the stimulatory effect was replaced by a decrease on DNA synthesis. Long-term exposure of MCF-7aro to **3a **and **4a **at the concentration range used resulted in an effective inhibitory effect on DNA synthesis. For instance, after 6 days of incubation, compound **3a **resulted in 18 ± 8%, 37 ± 10%, 50 ± 2% and 80 ± 3% and compound **4a **in 37 ± 5%, 52 ± 5%, 68 ± 3% and 86.5 ± 2% decrease in thymidine incorporation respectively for 1, 10, 25 and 50 μM. So compound **4a **showed to be slightly more effective in inhibiting MCF-7aro cell proliferation than **3a**.

### Cell cycle analysis

**Figure 6:**
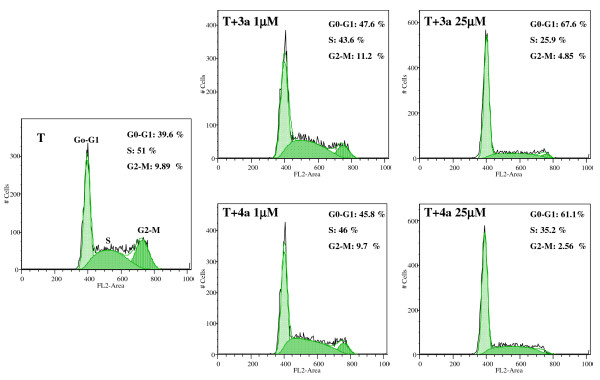
**Effects of compounds 3a and 4a on cell cycle distribution**. MCF-7aro cells were treated with 3a and 4a at 1 and 25 μM for 24 hr and subjected to flow cytometric analysis after PI staining. Compounds 3a and 4a induced cell cycle arrest in G_0_/G_1 _phase. Histograms were analysed with FlowJo Software (Tree Star, Inc). Data are representative of three independent experiments performed in triplicate.

To investigate the mechanisms underlying the antiproliferative effect of compounds **3a **and **4a **in MCF-7aro cells, discrimination of cells in G_0_/G_1 _versus S and G_2_/M phases of cell cycle was carried out by measuring DNA content by flow cytometry. Cells were treated with different concentrations of these two compounds and stained, after 24 hr, with PI. MCF-7aro cells deprived of hormones showed 91.3 ± 2.1% of cells in G_0_/G_1 _phase, whereas in testosterone-induced growth of MCF-7aro cells (control cells), the percentage of cells arrested in G_0_/G_1 _phase was only 42.4 ± 2.3%, which indicates that aromatase present in MCF-7aro is converting testosterone into estradiol, inducing in that way cell proliferation. The cell cycle profile of MCF-7aro cells treated with **3a **or **4a **was similar (Figure 6). Each treatment clearly increased the percentage of MCF-7aro cells in G_0_/G_1 _phase and reduced the fraction of cells in S and G_2_/M cell cycle phases (Figure 6 and Table 1). In response to treatment with **3a**, a dose-dependent accumulation of cells in G_0_/G_1 _phase was observed: 52.4 ± 6.8%, 53.6 ± 2.2%, 65.9 ± 2.1% and 72.2 ± 1.5%, respectively for 1, 10, 25 and 50 μM compared with 42.4 ± 2.3% in the control (Table 1A). Like steroid **3a**, compound **4a **presented a dose-dependent increase in the cell percentage in G_0_/G_1_: 58.6 ± 18.0%, 43.6 ± 1.4%, 59.6 ± 2.2%, 78.2 ± 2.3%, respectively for 1, 10, 25 and 50 μM (Table 1B). The same pattern of steroidal AIs effect on cell cycle progression were also observed after 3 days of treatment however no statistically significant differences was found.

**Table 1:** Effects of steroids 3a (A) and 4a (B) on cell cycle distribution of human breast cancer MCF-7aro cells.

***A***	*Control*	*T*	*T + 3a1 μM*	*T + 3a10 μM*	*T + 3a25 μM*	*T + 3a50 μM*
G_0_/G_1_	91.3 ± 2.1	42.4 ± 2.3 *	52.4 ± 6.8 ^φ^	53.6 ± 2.2 ^φ^	65.9 ± 2.1 ^φ^	72.2 ± 1.5 ^φ^
S	4.8 ± 1.2	48.4 ± 2.3 *	39.7 ± 5.5 ^φ^	38.3 ± 6.5 ^φ^	25.8 ± 3.3 ^φ^	19.6 ± 4.2 ^φ^
G_2_/M	5.0. ± 2.1	10.2 ± 2.4	9.94 ± 1.7	7.2 ± 4.6	7.4 ± 2.7	8.9 ± 2.6

***B***	*Control*	*T*	*T + 4a1 μM*	*T + 4a10 μM*	*T + 4a25 μM*	*T + 4a50 μM*

G_0_/G_1_	91.3 ± 2.2	42.4 ± 2.3 *	58.6 ± 18.0	43.6 ± 1.4	59.6 ± 2.2 ^φ^	78.2 ± 2.3 ^φ^
S	4.8 ± 1.2	48.4 ± 2.3 *	33.8 ± 17.3	43.4 ± 3.5	35.3 ± 0.1**	18.9 ± 1.8 ^φ^
G_2_/M	5.0 ± 2.1	10.2 ± 2.4	10.7 ± 1.4	13.1 ± 1.9	4.1 ± 2.2	3.0 ± 0.1

Cells were treated with different concentrations of the compounds for 24 hr. Treated cells were harvested, fixed and their DNA content was evaluated by PI labelling followed by flow cytometry analysis. Data are presented as single cell events in G0–G1, S and the G2-M phases of the cell cycle. The data represents means and SE of triplicates and are representative of three independent experiments. Significant differences between the control (medium without hormones) and medium containing 1 nM T are indicated by * (P < 0.0001); medium containing 1 nM T vs medium containing different steroid concentrations are indicated as φ (P < 0.001), ** (P < 0.01) and θ(P < 0.05).

### Analysis of cell death

**Figure 7:**
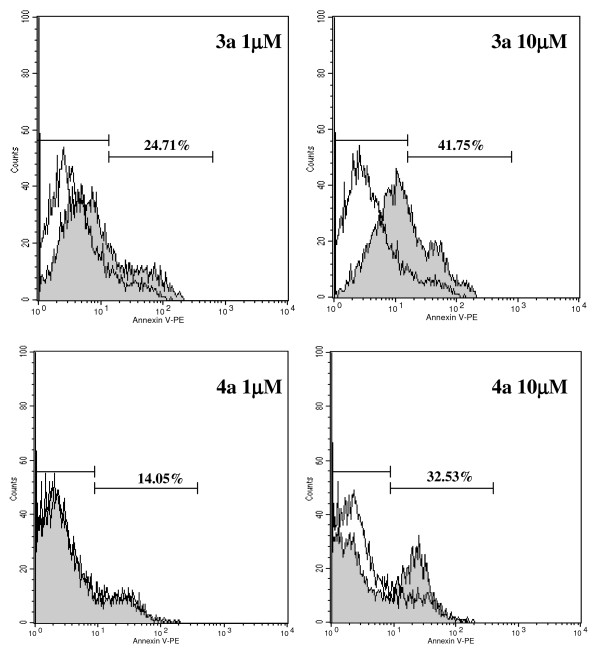
**Annexin V-PE labelling of MCF-7aro cells treated with 3a and 4a in comparison to control**. Cells were stained with annexin V-PE and 7-AAD and analysed by flow cytometry. Compounds 3a and 4a (gray filled histograms) induced an increase in annexin V binding in MCF-7aro cells, in comparison to 9.2% of the control (medium containing 1 nM T, open histograms). Numbers in histograms are percent of annexin V-positive cells after treatment with the compounds. The histograms correspond to cells gated for negative 7-AAD staining. Data are representative of triplicate cultures and the figure is representative of three independent experiments.

As shown previously (Figure 2, 3), incubation of MCF-7aro cells with compounds **3a **and **4a **caused alterations in cell morphology, such as membrane blebbing, chromatin condensation/marginalization and appearance of perinuclear vesicles in the cytoplasm. In order to investigate the type of cell death induced by these compounds, features associated with apoptosis or autophagic cell death were assessed. For apoptosis, plasma membrane changes such as phosphatidylserine (PS) residues in the outer surface of the plasma membrane were evaluated by annexin V-PE apoptosis detection kit. Staining with annexin V-PE was performed in association with a vital dye 7-amino-acitomycin (7-AAD), to allow the identification of viable, early apoptotic and late apoptotic or necrotic cells. Untreated MCF-7aro cells presented 9.2% binding to annexin V (Figure 7), whereas cells treated with **3a **at 1 μM and 10 μM showed 24.7% and 41.8%, respectively. Inhibitor **4a **induced an increased binding to annexin V of 14.05% and 32.53%, respectively for 1 μM and 10 μM in comparison to the control (9.2%). Caspases-3/7 activity was evaluated, but no changes were observed (data not shown) for both compounds at different incubation periods (6–96 hr).

**Figure 8:**
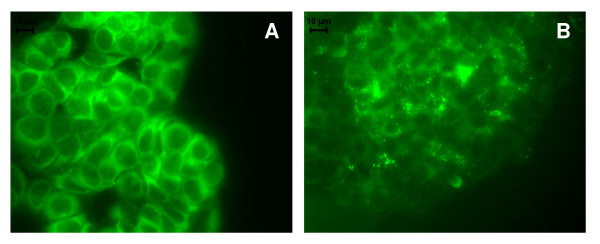
**Visualization of autophagic vacuoles with MDC in MCF-7aro cells**. MCF-7aro cells were incubated in the absence (A) or in the presence (B) of 25 μM of compound 4a in medium containing 1nM T. After 72 hr incubation with the compounds, cells were treated with MDC for 1 hr at 37°C, washed with PBS and analysed by fluorescence microscopy. The formation of autophagic vacuoles in MCF-7aro cells treated with compound 4a was indicated by punctuated MDC labelling in the cytoplasm.

**Figure 9:**
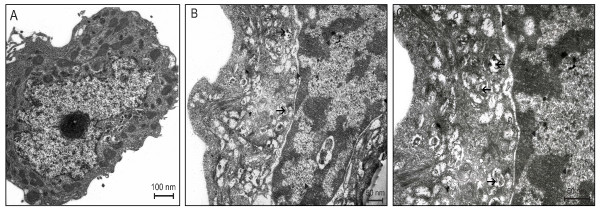
**Ultrastructural features of cell death in control MCF-7aro cells (A) and after treatment with 25 μM compound 4a (B-C) after 72 hr incubation**. (A) The cytoplasm tipically shows multiple polyribosomes and numerous mitochondria. (B) The cytoplasm shows multiple autophagic vacuoles containing cytoplasmic fragments (arrows). The chromatin is irregularly condensed (arrowheads). (C) is ahigher magnification of (B).

In order to clarify the nature of the cytoplasmic structures observed in the treated cells (Figure 2 and 3), the auto-fluorescent substance MDC, a dye for autophagic vacuoles, was used. In cells treated with compounds **3a **or **4a **(25 μM), MDC-labelled autophagic vacuoles appeared as distinct dot-like structures distributed in the cytoplasm or in the perinuclear regions whereas in the untreated cells, there was a homogeneous distribution of MDC (Figure 8). After 3 days, ultrastructural analysis demonstrated that the compounds induced an apoptotic-like nuclear morphology characterized by a partially condensed chromatin marginalized along the nuclear envelope (Figure 9). In addition, the cells presented giant autophagosomes distributed throughout the cytoplasm containing cytoplasmatic fragments and mitochondria.

## Discussion

Estrogens promote cell proliferation in normal and transformed mammary epithelial cells, activating hormone-responsive genes involved in the regulation of cell cycle. Studies have shown that anti-estrogenic treatment or estrogen deprivation prevents MCF-7 cells to enter the S and G_2_/M phase [13,28]. In addition to cell cycle progression, estrogens are known to protect cells against cell death by apoptosis. Several studies both *in vitro *and *in vivo *have reported that estrogen withdrawal [22] and treatment with either tamoxifen [23-25], faslodex [27] and some AIs [28] induce apoptosis of estrogen-dependent breast cancer cells. Anti-hormonal treatments of breast cancer are therefore central in regard to their potent inhibitory effects in hormone-induced cell growth.

This study explored the *in vitro *effects of compounds **3a **and **4a **in MCF-7aro cell growth, cell cycle progression and induction of cell death. In this system, MCF-7aro breast cancer cell line expressed sufficient aromatase activity in order to stimulate cell growth via aromatization of testosterone to estradiol. In fact, this androgen significantly stimulated MCF-7aro cells growth at concentrations as low as 1 nM, which is within the physiological concentration range for this steroid hormone. In the present work, we showed that compounds **3a **and **4a**, which have previously demonstrated to efficiently inhibit aromatase in placental microsomes [29] and in different cell lines [33], have also the ability to inhibit the proliferative action of testosterone in MCF-7aro cells in a dose- and time-dependent manner. This indicates that aromatization of the androgen with production of E_2 _was responsible for the testosterone-mediated cell growth. However, at 24 hr incubation, this effect was not observed. Indeed, inhibitors **3a **and **4a **induced stimulation of DNA synthesis, which might be due to estrogenic-like effects. A similar pattern of cell proliferation has been described for other compounds [34-37]. When assessing cell cycle it was found an increase in G_0_/G_1 _and a decrease in S and G_2_/M phases. We demonstrated that both aromatase inhibitors induce growth arrest of MCF-7aro cells by blocking the G_1_/S phase transition of cell cycle or a prolonged transit time in G_1_, preventing cells to enter S phase, which subsequently led to a decrease in the percentage of cells in S and G_2_/M phase. Moreover, thymidine incorporation results together with the fraction of cells in S phase suggest that cell proliferation may continue though with a reduction in growth rate. Analysis of cell cycle suggests that the mechanisms of action of both compounds are identical. These results are in accordance to a previous study [28] in which similar effects were induced by other AIs, like letrozole (Let), anastrozole (Ana), and 4-hydroxyandrostenedione (4-OH-A), in the cell cycle distribution of estrogen-dependent breast cancer cells.

We believe that the reduction in cell number induced by treatment of MCF-7aro cells with these inhibitors is due, not only to the prevention of cell proliferation by cell cycle arrest, but also to the induction of cell death. Thus, it is important to understand the mechanisms associated with cell death resulting from the treatment of ER-positive cells with inhibitors of aromatase. Our results showed that the treated cells presented morphological alterations typical of cells undergoing apoptosis, such as condensation/marginalization of chromatin and membrane blebbing, as well as vacuolization of the cytoplasm, a feature of autophagy. Typical apoptotic bodies were not observed. Previous studies revealed that MCF-7 cells are responsive to apoptotic stimuli [38] and although DNA fragmentation occurs [39], they lack pro-caspase-3 polypeptide [38]. Moreover, in previous published data using MCF-7 [39,40] and MCF-7aro cells [28], activation of caspase-3/7 was not observed for any of the treatments, which is in accordance to our results. This study also showed that compounds **3a **and **4a **induced increased binding of annexin V on MCF-7aro cells after 72 hr of treatment related with the phosphatidilserine exposure on the cell surface, which is known to have a key role on the clearance of apoptotic cells [41]. Nevertheless, Madden and colleagues [42] have shown that phosphatidylserine exposure, in addition to being a marker of apoptosis, can also occur in cells dying by autophagic cell death. Moreover, electron microscopy revealed the presence of many isolated autophagossomes engolfing cytoplasmic fractions and organelles. It has been reported that although features of various death pathways can be exhibited, only the most effective one is usually evident. In fact, autophagic cell death was induced in a promonocyte cell line treated with arsenic trioxide [43] and TGF-β induced both apotosis and autophagy in a mammary bovine cell line [43,44]. Electron microscopy results with **3a **and **4a**-treated MCF-7aro cells reinforce the origin of MDC-stained vesicles, showing the presence of multiple autophagosomes dispersed throughout the cytosol. It seems that different pathways of programmed cell death can be induced by these anti-aromatase treatments, ie. apoptosis and autophagic cell death, suggesting mutual interactions between these two types of cell death, however the molecular switch is not defined. Several studies pointed out that there is a crosstalk between apoptosis and autophagic cell death. Execution of apoptosis can be preceded by and may even depend on autophagy [45-47] or autophagy may rather antagonize or delay apoptosis and its inhibition may increase the sensitivity for the cells to apoptotic signals [48]. Motyl and colleagues have also shown that apoptosis might be accompanied by increased autophagy as a cytoprotective process, but in advanced stages might result in a particular type of programmed cell death. The threshold that separates these two processes is not clear and might depend on the extension of degradation of cellular components [44]. Some authors have previously shown that some AIs [28] induce cell death of breast cancer cell lines by apoptosis. However, it has also been reported that various anticancer therapies, including tamoxifen and other anti-estrogenic agents, induced autophagic cell death in breast cancer MCF-7 cells [49-52] and in FM3A breast cancer cells [53], and crotoxin can also induced this type of programmed cell death in MCF-7 cells [54].

## Conclusion

Our *in vitro *studies showed that the two steroidal AIs, **3a **and **4a**, are potent inhibitors of breast cancer cell proliferation. Moreover, it was also shown that the antiproliferative effects of these two steroids on MCF-7aro cells are mediated by disrupting cell cycle progression, through cell cycle arrest in G_0_/G_1 _phase and induction of cell death. We believe that both autophagic cell death and/or caspase-3 independent apoptosis may participate in MCF-7aro cell death induced by **3a **and **4a**, being the dominant mechanism autophagic cell death. The precise mechanisms underlying the different roles of autophagic cell death and the signalling pathways activated upon treatment of breast cancer cells with these steroidal AIs remain to be studied. In this way, it is important to understand the molecular components and signalling pathways of cell death machinery of cancer cells to help in the establishment of appropriate therapeutics, in order to provide new insights into the treatment of tumours with AIs.

## Methods

### Materials

Eagles's minimum essential medium (MEM), fetal bovine serum (FBS), L-glutamine, antibiotic-antimycotic 100× (10000 units/ml penicillin G sodium, 10000 mg/ml streptomycin sulphate and 25 mg/ml amphotericin B), Geneticin (G418) and trypsin were supplied by Gibco Invitrogen Co. (Paisley, Scotland, UK). Trypan blue, testosterone (T), ethylenediaminetetracetic acid (EDTA), dimethylsulfoxide (DMSO), sodium pyruvate, Hoeschst 33258, propidium iodide (PI), Triton X-100, DNase-free RNase A, monodansylcadaverine (MDC), staurosporine (STS), Wright stain, charcoal and dextran were from Sigma-Aldrich Co. (Saint Louis, USA). ^3^H-thymidine was supplied by Amersham (Amersham International, Amersham, UK). Caspase-GloTM -3/7 luminometric assay was from Promega Corporation (Madison, WI, USA) and Annexin V-PE apoptosis detection kit I was from BD Biosciences Pharmingen (San Diego, CA, USA). Liquid scintillation cocktail Universol was purchased from ICN Radiochemicals (Irvine, CA, USA). Fluorescence-activated cell sorter (FACS) buffer and FACS Rinse were from Becton Dickinson (San Jose, CA, USA). Vectashield mounting medium was from Vector (Burlingame, CA, USA). A detailed description of the preparation of the steroids studied in this work is published elsewhere [30]. A stock solution of 20 mM was prepared in 100% DMSO and stored as stock solutions at -20°C. Appropriate dilutions of the compounds were freshly prepared just prior to perform the assays.

### Preparation of charcoal-stripped fetal bovine serum (CFBS)

In order to avoid the interference of the steroids present in FBS and the estrogenic effects of phenol-red [31], four days before starting the experiments, MCF-7aro cells growing in standard MEM medium were changed to E_2_-free medium, which consisted of MEM medium without phenol-red with 5% pre-treated charcoal heat-inactivated fetal bovine serum (CFBS), 1 mmol/L sodium pyruvate, 2 mmol/L glutamine, 1% penicillin-streptomycin-amphotericin B and 700 ng/ml G418. Steroids were removed by incubating 500 ml of heat-inactivated FBS with 8 g activated-charcoal for 24 hr at RT, followed by centrifugation for 15 min at 4000 g to separate the charcoal from the serum. The supernatant was sterilized by passage through a cellulose acetate filter with a 0.22 μm pore size.

### Cell culture

The ER-positive aromatase-overexpressing human breast cancer MCF-7 cell line, MCF-7aro, prepared by stable transfection with the human placental aromatase gene and Geneticin selection [32], was kindly provided by Dr. Shiuan Chen (Beckman Research Institute, City of Hope, Duarte, CA, U.S.A.). Cells were maintained with Eagles's minimum essential medium (MEM) with Earle's salts and 1 mmol/L sodium pyruvate, 2 mmol/L glutamine, 1% penicillin-streptomycin-amphotericin B, 700 ng/ml G418 and 10% heat-inactivated fetal bovine serum in 5% CO_2 _at 37°C. Culture medium was changed every 3 days. At 80–90% confluence, cells were detached with 0.25% trypsin/1 mM EDTA during 1 min at room temperature. Cell morphology was studied by Wright staining.

### Thymidine incorporation assay

MCF-7aro cells were seeded in 96-well plates in a medium containing 5% CFBS and 1 nM testosterone (T) which was used as aromatase substrate and proliferation inducing agent. The cells were incubated with different concentrations of compounds **3a **and **4a **(1–50 μM). As a negative control, cells were incubated with culture medium plus DMSO. Incubations were maintained for 1–6 days and the medium and drugs were refreshed every 3 days. At each exposure time, ^3^H-thymidine (0.5 μCi) was added to each well and incubated at 37°C in 5% CO_2 _for the last 8 hr. After a cycle of freezing/defrosting, cells were harvested using a semi-automated cell harvester (Skatron Instruments, Norway), 1 ml scintillation cocktail was added and ^3^H-thymidine incorporated was determined in a scintillation counter (LS 6500, Beckman Instruments, CA, USA). Assays were carried out in triplicate and results are representative of at least three independent experiments.

### Cell cycle analysis

To investigate the antiproliferative effects of compounds **3a **and **4a **in MCF-7aro cells, cell cycle analysis was performed by flow cytometry. Cells (7 × 10^5^) were seeded in T-25 flasks and cultured in medium containing 1 nM T and with or without inhibitors **3a **and **4a**, at different concentrations (1–50 μM). Untreated cells, incubated with 1 nM T, were considered as control. After 24 hr of treatment, cells were harvested using 0.25% trypsin and 1 mM EDTA, mixed with non-adherent cells, washed twice with 5 ml PBS and resuspended in a final volume of 0.5 ml PBS. The cell suspension was transferred to 70% cold ethanol and kept at 4°C for ≥ 2 hr. The ethanol-suspended cells were centrifuged and the cell pellets washed in PBS. Fixed cells were finally resuspended in 0.5 ml DNA staining solution (5 μg/ml PI, 0.1% Triton X-100 and 200 μg/ml DNase-free RNase A in PBS) and kept 30 min at room temperature. Flow cytometric analysis of DNA content was based on the acquisition of 20000 events in a Becton Dickinson FACSCalibur (San Jose, CA, U.S.A) equipped with CELLQuest Pro software.

Debris, cell doublets and aggregates were gated out using a two parameter plot of FL-2-Area to FL-2-Width of PI fluorescence. Detectors for forward (FSC) and side (SSC) light scatter and the three fluorescence channels (FL-1, FL-2 and FL-3) were set on a linear scale. Cell cycle histograms were analysed using FlowJo Software (Tree Star, Inc). The antiproliferative effect of a compound in this assay is indicated by the percentage of cells in G_0_/G_1 _phase of the cell cycle. Assays were performed in triplicate.

### Analysis of apoptosis

Annexin V-PE apoptosis detection Kit was used, according to the manufacturer's instructions, to evaluate the apoptotic cells. Briefly, cells (2 × 10^5^) were cultured in 6-well plates and treated with or without inhibitors **3a **and **4a **at different concentrations (1–25 μM) for 72 h. Adherent and non-adherent cells after being pooled, washed and counted were incubated in binding buffer containing annexinV-PE (5 μl) and 7-ADD (5 μl) for 10 min at RT. As a positive control, cells were incubated for 12 hr with STS at 1 μM. Flow cytometric analysis was carried out in a FACS Calibur (San Jose, CA, U.S.A) based on the acquisition of 10000 events. Detectors for forward (FSC) and side (SSC) light scatter were set on a linear scale, whereas logarithmic detectors were used for all three fluorescence channels (FL-1, FL-2 and FL-3). Compensation for spectral overlap between FL channels was performed for each experiment using single-color-stained cell populations. All data were collected ungated to disk and were analyzed using CELLQuest Pro software.

Bivariant analysis of Annexin-PE fluorescence (FL-2) and 7AAD-fluorescence (FL-3) distinguished different cell populations. PE-ve and 7AAD-ve were designated as viable cells; PE+ve and 7AAD-ve were apoptotic cells and PE+ve and 7AAD+ve were considered as late apoptotic and necrotic cells.

Hoechst staining was used to evaluate alterations in nuclear morphology. After treatment for the indicated times and conditions, cells were washed twice with PBS, fixed with 4% paraformaldehyde in PBS (pH 7.4) for 10 min at RT, exposed to 0.5 mg/ml Hoechst 33258 in PBS for 20 min at room temperature and mounted in vectashield. The nuclear morphology was examined under a fluorescence microscope (Eclipse E400, Nikon, Japan), equipped with an excitation filter with maximum transmission at 360/400 nm, and processed by Nikon ACT-2U image software.

Caspase-3/7 activity was also evaluated using the Caspase-GloTM -3/7 luminometric assay, after incubation of MCF-7aro cells with the compounds for different incubation periods (6–96 hr).

### Analysis of intracellular vacuoles

MCF-7aro cells were seeded in 8-chamber wells and treated with compounds **3a **and **4a **(25 μM) for 72 hr. Fresh MEM containing 25 μM MDC was added to the cells and incubated at 37°C for 1 hr. After washing with PBS and mounting with vectashield medium, cells were immediately analysed in a fluorescence microscope (Axioskop, Carl Zeiss, Germany) equipped with a CCD Spot 2 camera (Diagnostic Instruments, USA) and image software Spot 3.1 (Diagnostic Instruments, USA). Images were obtained with a filter set 40 (Carl Zeiss, Germany) with excitation BP 360/51, beam splitter TFT 440+500+570 and emission filter TBP 460+520+600.

For electron microscopy, cells were harvested by tripsinization after 72 hr incubation with the compounds (25 μM), washed with sodium cacodilate (50 mM, pH 7.4), fixed with 1.25% glutaraldehyde/4% paraformaldehyde and preserved at 4°C for further processing. The cells were post-fixed in 1% osmium tetroxide in the same buffer, dehydrated in graded alcohols and embebed in Epon 812. Ultra-thin sections obtained with a Reichert Supra Nova ultramicrotome were collected on copper grids, stained with uranyl acetate/lead citrate and examined in a Zeiss 902A transmission electron microscope.

### Statistical analysis

The data presented are expressed as the mean ± SE. Statistical analysis of data was performed using analysis of variance (ANOVA) followed by Bonferroni post-hoc test for multiple comparisons. Values of P < 0.05 were considered as statistically significant.

## Authors' contributions

MC – Acquisition of data and analysis and interpretation of data; participation in manuscript's drafting GC–d–S – Analysis and interpretation of data; participation in manuscript's revision EJTdS – Synthesis of compounds and participation in manuscript's revision FMFR – Synthesis of compounds and participation in manuscript's revision MB – Analysis and interpretation of flow cytometry data NAT – Analysis and interpretation of data; participation in manuscript's revision and final approval
